# Family Material Hardship and Chinese Adolescents’ Problem Behaviors: A Moderated Mediation Analysis

**DOI:** 10.1371/journal.pone.0128024

**Published:** 2015-05-26

**Authors:** Wenqiang Sun, Dongping Li, Wei Zhang, Zhenzhou Bao, Yanhui Wang

**Affiliations:** 1 School of Psychology & Center for Studies of Psychological Application, South China Normal University, Guangzhou, China; 2 School of Psychology, Central China Normal University, Wuhan, China; 3 School of Educational Science, Jiaying University, Meizhou, China; University of Florida, UNITED STATES

## Abstract

In the current study, we examined a moderated mediation model using the risk and resilience framework. Specifically, the impact of family material hardship on adolescent problem behaviors was examined in a Chinese sample; we used the family stress model framework to investigate parental depression and negative parenting as potential mediators of the relation between family material hardship and adolescents’ problem behaviors. In addition, based on resilience theory, we investigated adolescents’ resilience as a potential protective factor in the development of their internalizing and externalizing problems. Participants included 1,419 Chinese adolescents (mean age = 15.38 years, *SD* = 1.79) and their primary caregivers. After controlling for covariates (age, gender, location of family residence, and primary caregiver), we found that parental depression and negative parenting mediated the association between family material hardship and adolescents’ problem behaviors. Furthermore, the adolescent resilience moderated the relationship between negative parenting and internalizing problems in a protective-stabilizing pattern; in addition, a protective-reactive pattern also emerged when adolescent resilience was examined as a moderator of the relationship between negative parenting and externalizing problems. These findings contribute to a comprehensive understanding of the mechanisms of risk and resilience in youth development. Moreover, the findings have important implications for the prevention of adolescent problem behaviors.

## Introduction

Problem behaviors among adolescents residing in impoverished conditions continue to be of concern to developmentalists and policy makers. There is a substantial amount of literature indicating that poverty and co-factors are risk factors for the development of internalizing and externalizing problems in adolescents [1–6]. However, not all adolescents living in poverty develop problem behaviors [7, 8]. Therefore, it is important to understand how and when poverty and co-factors operate as in the risk and protective processes.

The current study was conceptualized from the risk and resilience frameworks [9, 10]; specifically, we integrated the family stress model (FSM) [2, 11, 12] and resilience theory [13–16] to examine two research questions. First, based on the FSM, we examined proximal family risk factors (parental depression and negative parenting) as potential mediators of the relation between distal family risk factors (material hardship) and adolescent problem behaviors (internalizing and externalizing problems). Second, based on resilience theory, we examined adolescents’ resilience as a moderator of the relation between parental depression and negative parenting and adolescents’ internalizing and externalizing problems. The results of this study provide a better understanding of the risk and protective factors that influence the adjustment of Chinese adolescents residing in impoverished conditions, thereby offering valuable information about effective prevention and intervention methods [7, 9].

### Material hardship and adolescent problem behaviors

The majority of studies examining the relation between poverty and child development define poverty in terms of income; studies examining the influence of other dimensions of poverty, such as material hardship, are lacking [6]. Material hardship is a consumption-based indicator of economic well-being; it is based on the magnitude of financial hardship that families face, and includes indicators of the ability to pay monthly bills, buy food, and pay for shelter [17–19]. Empirical evidence has shown that the distributions of material hardship and income are not parallel; indeed, they are only moderately correlated [19–21]. Moreover, research has shown that families living in “near poor” households (with income ranging from 100% to 200% of the poverty threshold) also experienced one or more forms of material hardship, including not having enough food because of the inability to pay bills; thus, hardship is not limited to those living below the poverty line [18, 19, 20, 22]. Indeed, it is clear that measuring poverty via income has limitations. Therefore, a growing number of researchers have begun to use measures of material hardship to study the association between consumption patterns or basic standards of living and children’s developmental outcomes [17, 18, 23, 24, 25, 26]. Therefore, material hardship was used as an indicator of family economic constraint in the current study.

A standard measure of material hardship does not presently exist; however, many researchers have emphasized that one should be measured via indices of food availability, housing security, and the availability of medical care and financial conditions [18, 19, 20, 25]. Gershoff et al.’s method for assessing material hardship was followed in the present study; specifically, Gershoff and colleague measure material hardship via four domains: food insecurity, housing problems, financial trouble, and insufficient health care.

There are several empirical studies that indicate that material hardship has a negative impact on children’s problem behaviors. For example, these relations were examined in a nationally representative sample of children in the United States [18]; it was reported that material hardship was associated with lower levels of child social-emotional competence (including internalizing and externalizing problems). Similarly, in a longitudinal study, Zilanawala et al. [26] found that children residing in households experiencing material hardship scored significantly higher on internalizing and externalizing problems. These findings suggest that material hardship is an important predictor of children’s problem behaviors. Nonetheless, little is known about the mediating mechanisms underlying the relationship between material hardship and adolescents’ internalizing and externalizing problems.

### The mediating roles of parental depression and negative parenting

Recently, researchers have acknowledged that the link between economic hardship and children’s problem behaviors is likely mediated by several factors; specifically, proximal factors likely mediate the relationship between economic hardship and children’s problem behaviors [1, 3, 6, 27]. Indeed, Conger and colleagues [2, 11, 12] developed and tested the FSM of economic hardship. This model stipulates that economic hardship indirectly and adversely affects children’s developmental outcomes through its impact on parents’ psychological functioning (e.g., depression and anxiety) and behaviors (e.g., irritable, punitive, or rejecting parenting).

There is empirical support for this model among diverse racial and ethnic samples [2, 18, 24]. For instance, in a sample of African American families, Conger and colleagues [2] found that economic hardship was positively related to caregivers’ emotional distress; this was related to disrupted parenting practices that, in turn, predicted higher externalizing symptoms in children. Similarly, Mistry and colleagues [24] found that family stress processes were important mediators of the relationship between economic hardship and child behavior problems in a low-income, ethnically diverse sample. Therefore, it is clear that the FSM has been supported empirically across multiple studies.

However, these studies have exclusively focused on American children, who comprise less than 5% of the world’s population [28]. Indeed, evidence demonstrated that the link between socioeconomic status and child well-being varies as a function of geography and culture [1]. Because China is quite different from the United States in terms of economic and social security, families’ experiences and responses may differ. To date, very few studies have examined the impact of poverty or material hardship on parents’ well-being and children’s development with a Chinese population. In fact, approximately 11.8% of Chinese people (more than 100 million people) are living under the poverty line of annual income of RMB2,300 (about $375.55) [29]. Due to the large population in China, the number of adolescents living in poverty or near poverty is troubling. Therefore, in this study, we investigated the association between material hardship and adolescents’ problem behaviors in a Chinese sample. Based on the FSM and prior research, we expected that material hardship would indirectly impact adolescents’ internalizing and externalizing problems via parental depression and negative parenting.

### The moderating role of adolescents’ resilience

Despite exposure to multiple family risk factors, not all adolescents who live in impoverished settings develop problem behaviors; thus, it seems that certain individual and/or contextual factors may ameliorate the relationship between risk factors and adolescent problem behaviors [8, 9, 13, 14, 16]. Resilience is the dynamic process where an individual is able to adapt positively despite experiencing significant adversity. Therefore, resilience reflects a process of positive adaptation in the presence of risk that may be the result of individual factors, environmental factors, or the interplay between the two [14, 15]. A key aspect of resilience is the presence of both risk and protective factors that either contribute to positive outcomes or mitigate negative outcomes. Protective factors have been identified as assets that are reside within an individual (e.g., competence, coping skills, and affect regulation) or resources that are external to an individual (e.g., support from family members and others) [13, 16].

A risk-buffering model of resilience has been proposed to explain how protective factors operate to alter the trajectory from risk exposure to negative outcomes [13, 30]. Specifically, the model refers to processes where protective factors may mitigate the impact of risks on negative outcomes. This model is commonly tested via the interaction between a protective factor and a risk factor [8, 13, 16]. Protective factors interact with risk factors in several ways that influence adjustment, including protective-stabilizing and protective-reactive models. The protective-stabilizing model describes a pattern where the correlation between a risk factor and symptoms of maladjustment is significant when the level of a protective factor is low; however, the correlation is not significant when the protective factor is high. The protective-reactive model depicts a pattern where the correlation between a risk factor and symptoms of maladjustment is also significant when the level of a protective factor is low; however, the strength of the correlation is attenuated when the protective factor is high [13, 14]. In summary, resilience theory describes a conceptual model that explains how youth overcome adversity. Moreover, this theory can be used to enhance individuals’ strengths and help them build the positive aspects of their lives [13]. Importantly, there is empirical studies support for the protective models in studies of adolescent problem behaviors [31, 32]. For example, in a sample of urban, African-American youth, Li and colleagues [31] reported that youth confidence significantly interacted with poverty in a protective-stabilizing fashion in the prediction of both internalizing and externalizing symptoms; in addition, a protective-reactive pattern emerged when the interaction between chronic hassles and family support was examined as a predictor of youths’ externalizing symptoms.

While the protective models of resilience have been examined, there is limited research on the mechanisms that may ameliorate the relation between family poverty and children’s problem behaviors from the perspective of risk and resilience [6, 9, 10, 33]. In addition, most of the previous work in this area has examined the buffering effect of a single protective factor [31, 32]; however, researchers have paid increasingly more attention to the effects of cumulative protective factors given that they have more protective power than a single protective factor [34–36]. Therefore, in the present study, the cumulative protection of resilience on youth problem behaviors was tested by simultaneously examining individual power (goal planning, affect control, and positive thinking) and supportive power (family support and help-seeking). In summary, the current study expanded the FSM by examining the buffering effects of adolescents’ resilience on internalizing and externalizing problems.

### The Present Study

**Fig 1:**
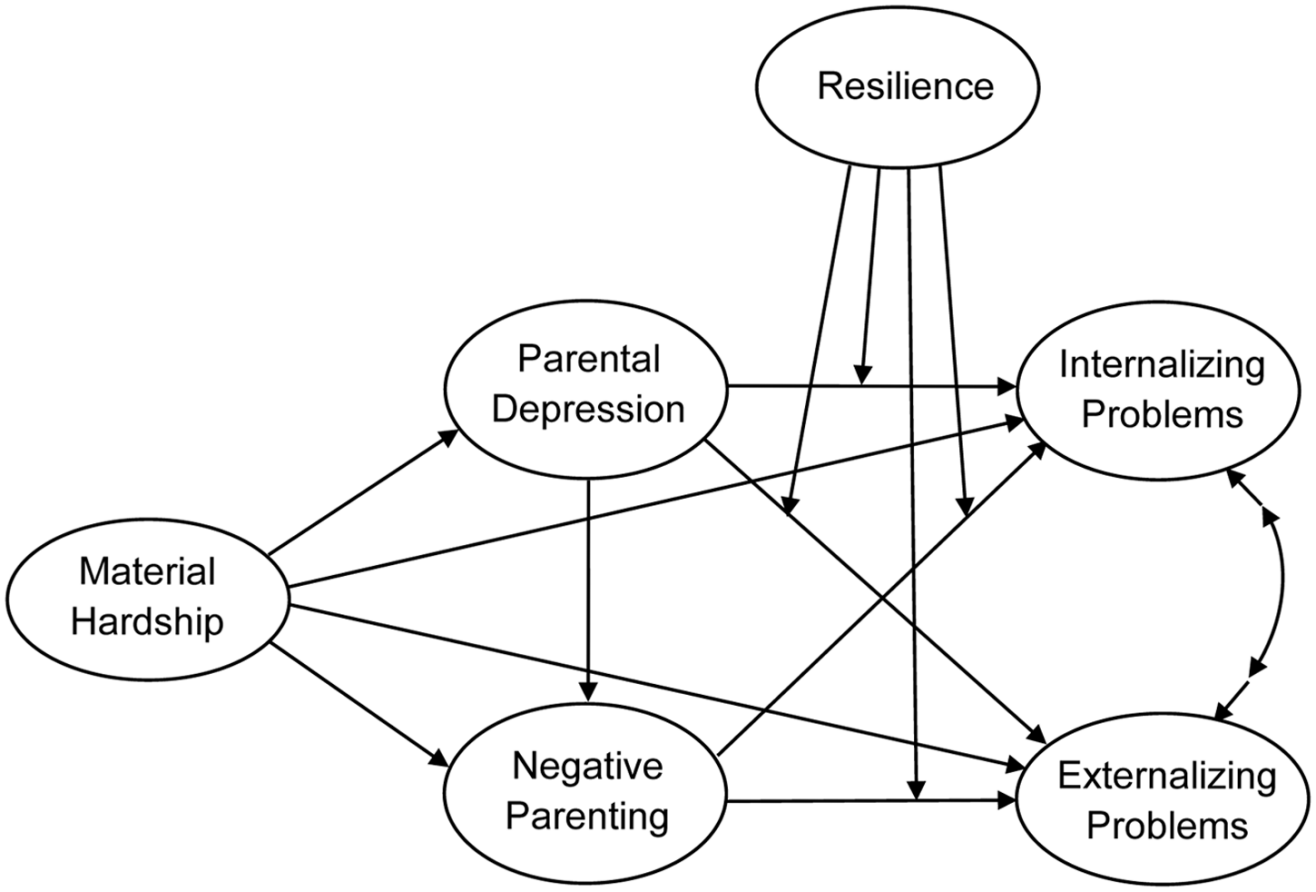
Conceptualized model with hypothesis paths.

In the current study, we merged two frameworks (FSM, resilience theory) and examined the mechanisms that underlie the relation between family material hardship and adolescents’ internalizing and externalizing problems. Specifically, it was our aim to build on the existing empirical research by simultaneously examining the mediating and moderating effects of family material hardship, parental depression, negative parenting, and resilience on adolescents’ internalizing problem behaviors. The conceptual model and the hypothesized paths are depicted in Fig 1. Specifically, based on the FSM, we hypothesized (Hypothesis A) that material hardship would be indirectly related to internalizing and externalizing problems mainly through its influence on parental depression and negative parenting. That is, when family material hardship was high, parents would be at an increased risk for depression; this increase would be related to more negative parenting behaviors that would lead to greater adolescent internalizing and externalizing problem behaviors. Moreover, the model including direct paths from material hardship to other variables would best fit the data.

We also examined hypotheses base on resiliency theory; specifically, we expected that resilience would buffer the effects of parental depression and negative parenting on internalizing and externalizing problems (Hypothesis B). These hypothesized relations represent a moderated mediation model [37], and the model proposes that the relationship between parental risks and adolescent problem behaviors is contingent on levels of adolescent resilience.

## Materials and Methods

### Ethics statement

The Human Research Ethics Committee of South China Normal University approved the research presented in this paper. All participants gave written, informed consent; they were informed of their right to discontinue participation at any time. Informed written consent was obtained from all adolescents and their primary caregivers; these procedures are consistent with the institutional guidelines of South China Normal University.

### Participants

Participants were recruited from six public middle schools (three junior high schools and three high schools) in northern and southern China. The consent rate was above 95% in the participating classrooms for adolescents and parents. After invalid questionnaires were eliminated (less than 3%), the total 1,419 adolescents (51% males) and their primary caregivers (58.8% of respondents are fathers, 37.6% of respondents are mothers and 3.7% of respondents are other caregivers) were used for the analysis. Adolescents mean age was 15.38 years (*SD* = 1.79). The majority of fathers (63.2%) and mothers (71.5%) did not complete high school; 10.8% of the fathers and 14.9% of the mothers reported that they did not have a full-time job during the past year. The education levels of the parents were similar to those of the local and national populations reported in the 2010 Chinese census data [38]. In addition, 21.8%, 16.2% and 62.0% of the students came from urban, suburban, and rural areas, respectively.

The data are available from S1 File.

### Measures

#### Family material hardship

Primary caregivers reported their family material hardship (food insecurity, housing problems, financial troubles, and insufficient health care) over the past 12 months [18, 19, 39].

Food insecurity was measured with the 18-item Core Food Security Module [40, 41]. Six of the questions were rated on a 3-point scale ranging from 0 = *never true* to 2 = *often true* (e.g., “We worried whether our food would run out before we got money to buy more”). The items were then dichotomized (0 = *never*; 1 = *sometimes or often true*) for the data analysis; this is consistent with prior studies [25, 42]. In addition, six questions were rated *yes* = 1 or *no* = 2 (e.g., “Did you ever eat less than you felt you should because there wasn’t enough money for food?”); these items were recoded (0 = *no*, 1 = *yes*), so that higher scores reflected increased food insecurity. There are also three preliminary questions that asked respondents whether they had skipped meals or eaten less at meals (e.g., “Did you or other adults in the household ever cut the size of your meals or skip meals because there wasn’t enough money for food?”); participants responded either *yes* = 1 or *no* = 0. If they answered *yes* to any of these questions, they were asked to respond to a follow-up question that asked them to rate how often they had cut/skipped meals for financial reasons. The follow-up question was rated on a 3-point scale ranging from 0 = *only 1 or 2 months* to 2 = *almost every month*; the responses to these questions were also dichotomized (0 = *only 1 or 2 months*; 1 = *some months but not every month or almost every month*) for the data analysis. The responses were averaged, with higher scores representing greater food insecurity. The Cronbach’s α for the present sample was 0.87.

Primary caregivers also indicated whether the family had lived in crowded conditions during the past year (0 = *no*, 1 = *yes*); if primary caregivers answered *yes*, they were asked to rate the frequency on a 3-point scale ranging from 0 = *only 1 or 2 months* to 2 = *almost every month*. This question was dichotomized (0 = *only 1 or 2 months*; 1 = *some months but not every month* or *almost every month*) during the data analysis. Primary caregivers were also asked if they had any maintenance problems in their home during the past year (e.g., “Problems with pests such as rats, mice, roaches, or other insects,” or “Broken window glass or windows that can’t shut.”); this item was rated 0 = *no* or 1 = *yes*. The responses were averaged across the three items, with higher scores representing greater housing problems.

In addition, primary caregivers indicated whether the family had serious financial problems, or if they were unable to pay their monthly bills in the past year (0 = *no*, 1 = *yes*); if they answered *yes*, primary caregivers then rated the frequency of financial problems on a 3-point scale ranging from 0 = *only 1 or 2 months* to 2 = *almost every month*. Their responses were dichotomized (0 = *only 1 or 2 months*; 1 = *some months but not every month or almost every month*) during the data analysis. The responses were averaged across the two items, with higher scores representing greater financial trouble.

Two items measured insufficient health were combined [19, 23]; each item was rated as 0 = *no* or 1 = *yes*. One item asked, “Was there anyone in your household who needed to see a doctor or go to the hospital but couldn’t because of the cost?” The other item asked, “Was there anyone in your household who needed to see a dentist but couldn’t because of the cost.” The responses were averaged across the two items, with higher scores representing greater insufficient health care.

The mean scores of food insecurity, housing problems, financial trouble and insufficient health care were used as the four manifest indicators of the material hardship latent variable. Confirmatory factor analysis indicated that the four-factor model demonstrated a good fit to the data: χ^2^(269, *N* = 1,419) = 1508.830, CFI = .978, TLI = .975, RMSEA = .057, a 90% RMSEA confidence interval [.054, .060]. The Cronbach’s α for material hardship was .89 in this sample.

#### Primary caregivers’ depression

Primary caregivers reported their feelings of depression using the Center for Epidemiological Studies Depression Scale (CES-D) [43]. The CES-D Scale is a 20-item self-report measure designed to measure symptoms of depression within the last week; the measure was designed for use in non-clinical, adult samples. Respondents provided ordinal responses ranging from 0 (*never or barely*) to 3 (*most or all of the time*). The measure yields four factors: depressed affect (“felt sad” or “crying spells”), positive affect (“felt happy” or “hopeful about future”), somatic and retarded activity (“appetite poor” or “restless sleep”) and interpersonal problems (“people dislike me” or “people were unfriendly”). The items from the positive affect factor were reverse scored. Adequate test-retest reliability and construct validity have been reported for this measure [43, 44]. Confirmatory factor analysis indicated that the four-factor model demonstrated a good fit to the data: χ^2^(162, *N* = 1,419) = 623.175, CFI = .937, TLI = .926, SRMR = .034, RMSEA = .045, a 90% RMSEA confidence interval [.041, .049]. The mean score of each factor was calculated and served as four manifest indicators of the parental depression latent variable. The Cronbach’s α for depression was .85 in the present study.

#### Negative parenting behaviors of primary caregivers

Primary caregivers completed the 12-item authoritarian parenting scale from the Parenting Style and Dimensions Questionnaire (PSDQ) [45]. The twelve items reflect physical coercion (e.g., “I slap my child when the child misbehaves”), verbal hostility (e.g., “I explode in anger toward my child”) and non-reasoning/punitive behavior (e.g. “I use threats as a punishment with little or no justification”). All items were rated on a 5-point scale ranging from 1 = *never* to 5 = *always*. Adequate test-retest reliability and construct validity have been reported with this measure; it has been widely used in Chinese samples [45–47]. Confirmatory factor analysis indicated that the three-factor model adequately fit the data: χ^2^(51, *N* = 1,419) = 262.571, CFI = .952, TLI = .938, SRMR = .035, RMSEA = .054, a 90% RMSEA confidence interval [.048, .061]. The mean score of each factor was calculated and served as three manifest indicators of the negative parenting latent variable. The Cronbach’s α of negative parenting was .84 in the present study.

#### Resilience

Adolescent resilience was measured with the 27-item Resilience Scale for Chinese Adolescents [48]. This questionnaire assesses five aspects of resilience: (1) goal planning (e.g., “I have a definite goal in my life”); (2) affect control (e.g. “Failure always makes me discouraged”); (3) positive thinking (e.g., “Adversity is helpful for growth”); (4) family support (e.g., “Parents always like to interfere with my ideas”); and (5) help-seeking (e.g. “When I’m in a difficult situation, I can’t find some people to rely on”). In addition, goal planning, affect control and positive thinking reflect the higher-order factor of individual power; help-seeking and family support reflect the higher-order factor of supportive power. Participants rated each statement on a 5-point scale ranging from 1 = *completely disagree* to 5 = *completely agree*. Adequate test-retest reliability and construct validity have been reported for this measure [48, 49]. Higher-order confirmatory factor analysis indicated that the model adequately fit to the data: χ^2^(311, *N* = 1,419) = 1304.850, CFI = .898, TLI = .885, SRMR = .052, RMSEA = .047, a 90% RMSEA confidence interval [.045, .050]. The mean score of each higher-order factor was calculated; these two factors served as two manifest indicators of the adolescent resilience latent variable. The Cronbach’s α for resilience was .86 in the present sample.

#### Internalizing problems

Adolescent internalizing problems were measured with the 32-item internalizing problems scales of the Youth Self-Report (YSR) [50, 51]. Adolescents reported their internalizing problems over the past 6 months on a 3-point Likert scale (0 = *not true*, 1 = *somewhat or sometimes true*, and 2 = *very true or often true*). The YSR internalizing scale comprises three subscales: withdrawal (e.g., “underactive” or “secretive”), somatic complaints (e.g., “feels dizzy” or “aches, pains”), and anxiety/depression (e.g., “nervous, tensed” or “feels worthless”). Adequate test-retest reliability and construct validity have been reported for this scale [51, 52]. Confirmatory factor analysis indicated that the three-factor model demonstrated good fit to the data: χ^2^(244, *N* = 1,419) = 988.867, CFI = .930, TLI = .921, SRMR = .039, RMSEA = .046, a 90% RMSEA confidence interval [.043, .049]. The mean score of each factor was calculated and served as three manifest indicators of the internalizing problems latent variable. The Cronbach’s α for internalizing problems was .91 in the present study.

#### Externalizing problems

Adolescent externalizing problems were measured with the 28-item externalizing problems scales of the YSR [50, 51]. Adolescents rated their externalizing problems over the past 6 months on a 3-point Likert scale (0 = *not true*, 1 = *somewhat or sometimes true*, and 2 = *very true or often true*). The YSR externalizing scale comprises two subscales: delinquent behaviors (e.g., “swearing, obscene language” or “steals outside home”) and aggressive behaviors (e.g., “destroys own things” or “attacks people”). Adequate test-retest reliability and construct validity have been reported [51, 52]. Confirmatory factor analysis indicated that the two-factor model adequately fit the data: χ^2^(254, *N* = 1,419) = 1129.038, CFI = .887, TLI = .867, SRMR = .049, RMSEA = .049, a 90% RMSEA confidence interval [.046, .052]. The averaged score of each factor was calculated and served as the two manifest indicators of the externalizing problems latent variable. The Cronbach’s α for externalizing problems was .87 in the present sample.

#### Covariates

Given that prior research has indicated that internalizing and externalizing problems varied by adolescent gender and age [53, 54], these variables were included as covariates in the model. In addition, participant residence (urban, suburban and rural) and primary caregiver (father, mother or others) were also included as covariates; these covariates were dummy coded into two variables with city and father as the reference categories, respectively.

### Procedure

Adolescents provided their primary caregivers with an explanatory statement and consent form. Parents who provided consent for their child to participate were required to return a signed consent form. Adolescents were also asked to complete a consent form according to the requirement of the ethics committee. The student-reported questionnaires were administered in classrooms by trained graduate students. The survey administrators explained the requirements and the confidentiality procedures to all participants; the survey administrators also monitored survey completion. Students were given approximately 20 minutes to complete the questionnaires during class time; parent-report questionnaires were completed at home and were returned to the school in the following morning.

### Analysis plan

There were small, non-significant intraclass correlations (ICC) among school levels for the internalizing problems (ICC = 0.082, Wald *Z* = 1.504, *p* = 0.133) and externalizing problems (ICC = 0.047, Wald *Z* = 1.446, *p* = 0.148). Therefore, the analyses were performed at an individual level [55].

The data were analyzed in the following steps. First, the expectation-maximization (EM) algorithm [56] in SPSS 20 was used to impute missing data; this procedure was appropriate given that the rate of missing scale items was less than 3.7% (the overall average was 0.39%). Second, structural equation modeling (SEM) was then performed in two steps using Mplus 7 [57]. Specifically, confirmatory factor analysis was conducted to evaluate the measurement model; then, SEM modeling was performed to test the mediating effects of parental risks (parental depression and negative parenting) and moderating effects of adolescent resilience with the maximum likelihood estimation. Since some of the latent variable indicators were not normally distributed (e.g., delinquency, interpersonal problems), bootstrap analysis was used to test the significance of the indirect effects. This calculation was repeated with 5,000 samples to yield a parameter estimate of both the total and specific indirect effects. If the 95% bias-corrected confidence interval for the parameter estimate was not contain zero, then the indirect effect was statistically significant, indicating a mediating effect [58]. In addition, the latent moderated structural equation (LMS) method was used to test the latent variable interaction [59]. In this procedure, a significant nonzero product term indicates the presence of an interaction. Simulation studies have demonstrated that the standard error estimates of LMS remain relatively unbiased even when some variables are non-normal [60].

Model fit was assessed using multiple fit indices, including the comparative fit index (CFI), the Tucker-Lewis Index (TLI), the root mean square error of approximation (RMSEA), and the standardized root mean square residual (SRMR). The model fit is considered adequate when CFI and TLI values are greater than .90, and the RMSEA and SRMR values are less than .08 [61, 62].

However, when specifying a latent interaction term in a model, these model fit indices cannot be estimated because of a lack of a comparative model [63]. We followed the procedure described by Perren et al. [64]. Specifically, two models were tested. The first model was tested without the latent variable interaction (restricted model); the second model with the latent variable interaction (full model) was then tested. The nested model Likelihood Ratio Test [LR = -2 × (LogL_Restricted_ - LogL_Full_)] was used to compare the difference between the two models. If the first model had adequate overall model fit and the LR test found that adding latent variable interaction significantly improved model fit, then we could conclude that the second model had an adequate model fit. In addition, the metrics of latent variables were set by fixing their variances at one, and the mean of each of the latent variables was fixed at zero by LMS [65] and LMS only offered an unstandardized solution.

In the mediating and moderating analysis, all of the latent variable indicators were centered to minimize multi-collinearity. Adolescent age, gender, primary caregiver, and family location were also included as covariates in the SEM analyses.

## Results

### Descriptive and preliminary analyses

The means, standard deviations, and correlations of all variables are displayed in Table 1. Generally, indicators were related to one another within and across constructs; the correlations were in the expected directions. For example, all of the indicators of material hardship were significantly and positively correlated with the indicators of parental depression and negative parenting. Similarly, the indicators of material hardship, parental depression, and negative parenting were significantly correlated with at least one of the indicators of adolescents’ internalizing and externalizing problems. The indicators of resilience were negatively associated with indicators of parental depression, negative parenting, and adolescents’ internalizing and externalizing problems.

**Table 1:** The Correlations Between the Study Variables.

Variables	1	2	3	4	5	6	7	8	9	10	11	12	13	14	15	16	17	18	19	20	21	22	23	24
**1. Age**	—																							
**2. Gender**	-.*01*	—																						
**3. Mother**	-.07	-.12	—																					
**4. Other**	.07	.*01*	-.15	—																				
**5. Suburban**	.11	-.*00*	-.*02*	.*01*	—																			
**6. Rural**	.*04*	.*03*	-.09	.*01*	-.56	—																		
**7. Food Insecurity**	.14	-.*05*	-.*04*	.*02*	-.08	.22	—																	
**8. Housing Problems**	.11	-.*05*	-.02	.06	-.*05*	.18	.49	—																
**9. Financial Trouble**	.12	-.06	-.06	-.*00*	-.*04*	.20	.51	.45	—															
**10. Insufficient Health Care**	.11	-.*04*	.*01*	.*03*	-.*03*	.07	.44	.37	.39	—														
**11. Depressed Affect**	.19	-.06	.07	.*04*	-.*01*	.08	.41	.31	.35	.35	—													
**12. Positive Affect**	.04	-.06	.*00*	-.*01*	.*04*	.*01*	.19	.11	.11	.16	.29	—												
**13. Somatic and Retarded Activity**	.14	-.*01*	.*05*	.*03*	-.*02*	.08	.41	.31	.35	.35	.74	.22	—											
**14. Interpersonal Problems**	.09	.*01*	-.*01*	.06	-.*01*	.*04*	.23	.20	.18	.16	.53	.25	.46	—										
**15. Physical Coercion**	.00	.08	.*03*	.*04*	.*00*	-.*04*	.18	.18	.15	.16	.30	.16	.29	.21	—									
**16. Verbal Hostility**	.03	.*02*	.06	-.*00*	.*04*	-.10	.11	.12	.12	.14	.26	.12	.28	.16	.58	—								
**17. Non-reasoning/Punitive**	.06	.08	.*00*	.*05*	.*04*	-.*05*	.17	.13	.11	.13	.32	.18	.33	.25	.55	.54	—							
**18. Individual Power**	-.13	.06	-.*02*	.*02*	-.*04*	.07	-.11	-.12	-.06	-.16	-.20	-.21	-.17	-.11	-.12	-.14	-.14	—						
**19. Supportive Power**	-.09	-.11	-.*00*	-.*00*	-.*04*	.07	-.11	-.10	-.*05*	-.11	-.16	-.17	-.19	-.12	-.23	-.25	-.27	.50	—					
**20. Withdrawal**	.29	-.*05*	.*03*	.*03*	.*02*	.*03*	.16	.13	.12	.18	.28	.11	.26	.17	.14	.19	.18	-.37	-.40	—				
**21. Somatic Complaints**	.13	-.12	.07	-.*02*	.*02*	-.*00*	.16	.14	.12	.22	.26	.15	.26	.15	.18	.16	.21	-.30	-.26	.50	—			
**22. Anxiety/Depression**	.18	-.09	.*02*	.*03*	.*02*	-.*02*	.15	.09	.11	.18	.29	.14	.27	.19	.18	.20	.24	-.46	-.42	.71	.58	—		
**23. Delinquency**	.13	.12	.*02*	.*02*	.*03*	-.07	.15	.06	.*03*	.12	.24	.14	.21	.22	.22	.21	.28	-.29	-.29	.38	.35	.44	—	
**24. Aggression**	.17	.*00*	.*02*	.*03*	.*03*	-.06	.15	.10	.10	.18	.26	.14	.24	.20	.22	.24	.28	-.39	-.32	.52	.47	.66	.62	—
***M***	15.38	.49	.38	.04	.*16*	.62	.15	.24	.22	.23	.35	1.04	.52	.20	1.70	2.29	1.64	3.56	3.53	.58	.42	.52	.15	.44
***SD***	1.79	.50	.48	.19	.*37*	.49	.19	.29	.34	.35	.44	.81	.46	.44	.64	.77	.65	.58	.70	.36	.34	.37	.19	.27

Note. N = 1,419. Gender was dummy coded: 0 = female and 1 = male. Primary caregiver was dummy coded into mother (= 1) and other (= 1), with father (= 0) as the reference category. Family location was dummy coded into suburban (= 1) and rural (= 1), with urban (= 0) as the reference category. Correlations were significant at the p < .05; the italicized values indicate that the correlations were no-significant at (p > .05).

In addition, the prevalence of internalizing and externalizing problems according to the clinically meaningful threshold (higher than 2 SD) [66] was 3.7% and 4.2%, respectively. In the present sample, 78.6% family experienced one or more of the indicators of material hardship.

### Measurement model test

Prior to running the SEM analyses to test the hypotheses, a measurement model was first established for all latent variables using confirmatory factor analyses [61]. The model fit the data well: χ^2^(120, *N* = 1,419) = 423.898, CFI = .968, TLI = .960, SRMR = .036, RMSEA = .042, a 90% RMSEA confidence interval [.038, .047]. The standardized factor loadings of each indicator were significant on their corresponding factors at *p* < .001 (see Table 2). The pattern of intercorrelations among the latent variables is shown in Table 3. Higher levels of family material hardship were associated with higher levels of parental depression, negative parenting, and internalizing problems, and externalizing problems. Higher levels of parental depression were associated with higher levels of negative parenting, and internalizing problems, and externalizing problems. Higher levels of negative parenting were associated with higher levels of internalizing and externalizing problems. Adolescents’ resilience was negatively associated with material hardship, parental depression, negative parenting, internalizing problems, and externalizing problems.

**Table 2:** The Measurement Model: Latent Variable Factor Loadings.

Variables	Standardized loading coefficients
**Material Hardship**	
Food insecurity	.80***
Housing problems	.62***
Financial trouble	.72***
Insufficient health care	.57***
**Parental Depression**	
Depressed affect	.89***
Positive affect	.32***
Somatic and retarded activity	.83***
Interpersonal problems	.58***
**Negative Parenting**	
Physical coercion	.76***
Verbal hostility	.74***
Non-reasoning/Punitive	.74***
**Resilience**	
Individual power	.72***
Supportive power	.72***
**Internalizing Problems**	
Withdrawal	.78***
Somatic complaints	.64***
Anxiety/Depression	.92***
**Externalizing Problems**	
Delinquency	.67***
Aggression	.93***

*Note*.

***p < .001.

**Table 3:** Correlation Matrix for the Latent Variables.

Latent Variables	1	2	3	4	5	6
**1. Material Hardship**	—					
**2. Parental Depression**	.58***	—				
**3. Negative Parenting**	.27***	.46***	—			
**4. Resilience**	-.19***	-.29***	-.35***	—		
**5. Internalizing Problems**	.22***	.38***	.30***	-.67***	—	
**6. Externalizing Problems**	.20***	.33***	.37***	-.54***	.76***	—

*Note*.

***p < .001.

### Testing the mediating effects of parental depression and negative parenting

The 95% bias-corrected bootstrap confidence intervals were used to test the significance of the direct, indirect, and total effects in the mediation model [33, 58, 67] since some of the latent variable indicators were not normally distributed (e.g., delinquency, interpersonal problems). In step 1, the baseline model (covariates and material hardship) was used to examine the direct effect of material hardship on adolescent’s problem behaviors. The model fit the data well: χ^2^(66, *N* = 1,419) = 366.521, CFI = .940, TLI = .919, SRMR = .049, RMSEA = .057, a 90% RMSEA confidence interval [.051, .062]; this indicated that material hardship significantly and positively predicted adolescents’ internalizing and externalizing problems, with 9.6% and 8.5% of the variance explained for internalizing and externalizing problems, respectively. In step 2, negative parenting was added to the baseline model (the order in which negative parenting and parental depression were added to the baseline model did not influence the final model). This model also demonstrated a good fit to the data: χ^2^(108, *N* = 1,419) = 495.396, CFI = .941, TLI = .924, SRMR = .046, RMSEA = .050, a 90% RMSEA confidence interval [.046, .055], thereby indicating that negative parenting partially mediated the effects of material hardship on adolescents’ internalizing and externalizing problems. The variance explained in the prediction of negative parenting, internalizing problems and externalizing problems was 7.4%, 16.1% and 17.2%, respectively. In step 3, parental depression was added to the model (saturated model) based on step 2. This model also fit the data well: χ^2^(178, *N* = 1,419) = 679.835, CFI = .944, TLI = .932, SRMR = .047, RMSEA = .045, a 90% RMSEA confidence interval [.041, .048]. The percent variance accounted for in the prediction of parental depression, negative parenting, internalizing problems, and externalizing problems was 34.1%, 21.1%, 19.2% and 18.6%, respectively. However, path coefficients also indicated some non-significant links. Therefore, in order to develop the most parsimonious model, we eliminated the non-significant paths in the sequence of the saturated model and tested for differences in model fit. In the final model, we eliminated three paths: material hardship to internalizing problems, material hardship to externalizing problems and material hardship to negative parenting. The difference between the full model and final model was non-significant, Δχ^2^(3, *N* = 1,419) = 1.124, *p* = .771, suggesting that the full model offered no additional explanatory power over the more parsimonious one. The final model was found to fit the data well, χ^2^(181, *N* = 1,419) = 680.959, CFI = .944, TLI = .934, SRMR = .047, RMSEA = .044, a 90% RMSEA confidence interval [.041, .048]. The amount of variance accounted for in the prediction of parental depression, negative parenting, internalizing problems and externalizing problems was 34.2%, 21.2%, 19.2% and 18.6%, respectively.

**Fig 2:**
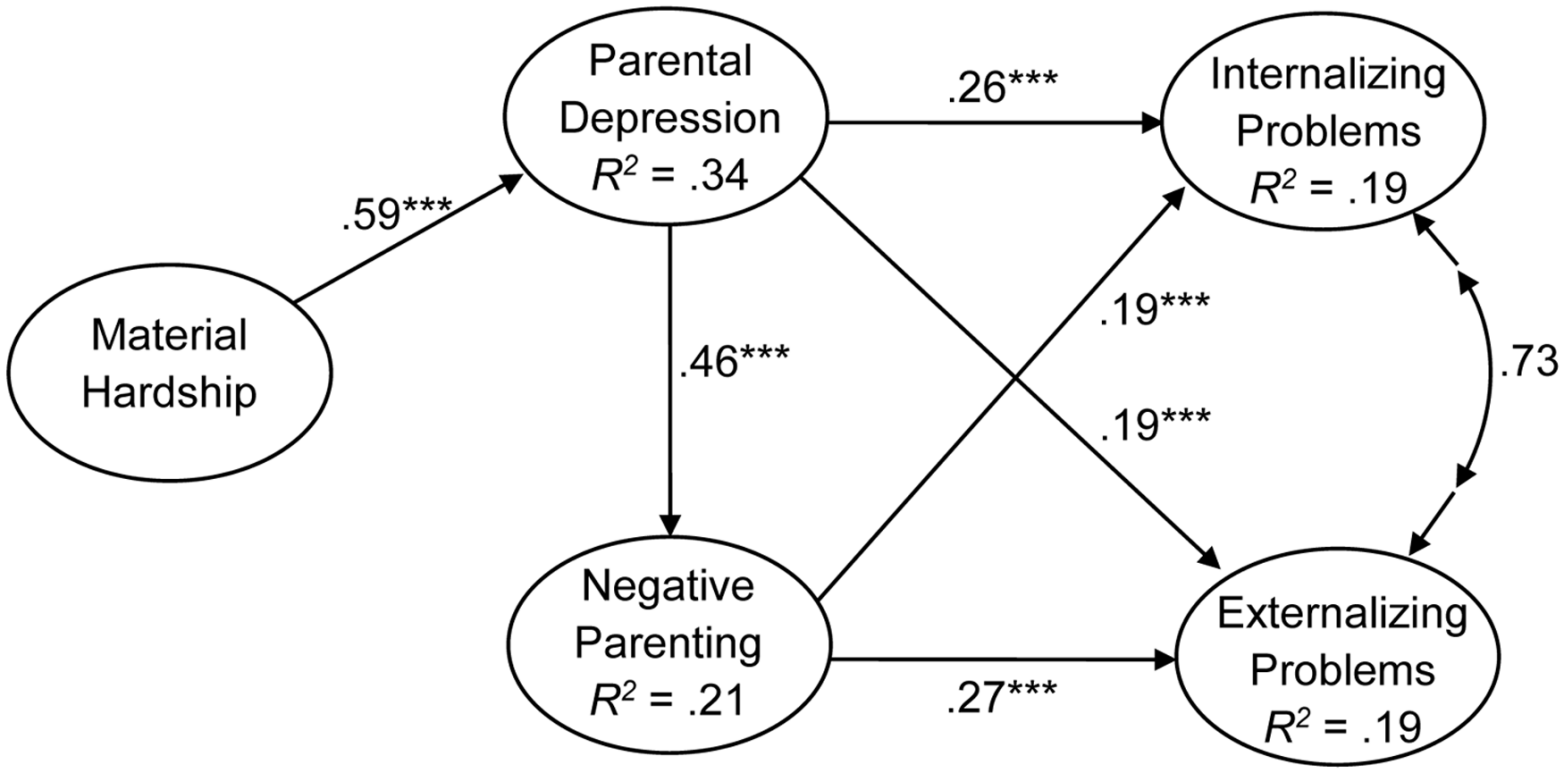
Standardized path estimates for parsimonious model with mediated effects. Age, gender, primary caregiver, and family location were included as covariates variable in this model, but is not shown in this figure. Curved arrows between internalizing and externalizing problems represent correlated errors. ***p < .001.

As shown in Fig 2, material hardship had a significant direct effect on parental depression (β = .59, *p* < .001), parental depression had a significant direct effect on negative parenting (β = .46, *p* < .001), and negative parenting had significant direct effects on internalizing problems (β = .19, *p* < .001) and externalizing problems (β = .27, *p* < .001). Parental depression had significant direct effects on internalizing problems (β = .26, *p* < .001) and externalizing problems (β = .19, *p* < 0.01). In addition, the main effect of gender (β = -.22, *p* < .001) was significantly and negatively associated with internalizing problems, indicating that internalizing problems were more common among females than males. The main effects of age was also significantly associated with internalizing problems (β = .12, *p* < .001) and externalizing problems (β = .10, *p* < .001), indicating that older children experienced more problem behaviors.

Material hardship did not have a significant direct effect on internalizing problems, but there was a significant indirect effect (material hardship → parental depression → internalizing problems, β = .15, *p* < .001; material hardship → parental depression → negative parenting → internalizing problems, β = .05, *p* < .001); and total effects (β = .20, *p* < .001). There was not a significant direct effect for the link between material hardship and externalizing problems; however, the indirect effect was significant (material hardship → parental depression → externalizing problems, β = .11, *p* < .001; material hardship → parental depression → negative parenting → externalizing problems, β = .07, *p* < .001); and total effects (β = .18, *p* < .001). These results partially supported our first hypothesis (Hypothesis A).

### Testing the moderating effect of adolescents’ resilience

To test the hypothesized moderating effects of adolescents’ resilience, two models were estimated. In the first model, the main effects of resilience in the prediction of internalizing and externalizing problems were added to the mediation model. This model had adequate model fit: χ^2^(221, *N* = 1,419) = 975.697, CFI = .925, TLI = .911, SRMR = .058, RMSEA = .049, a 90% RMSEA confidence interval [.046, .052]. The percent variance accounted for in the prediction of internalizing problems and externalizing problems improved to 46.7% and 32.3%, respectively. A second model was specified by adding two latent interaction effects (Parental Depression × Resilience, Negative Parenting × Resilience) to the first model. Likelihood ratio tests (the test statistic of the Loglikelihood is a distributed chi-square where the degrees of freedom are equal to the difference in the number of model parameters) were used to compare whether inclusion of the interaction terms improved the model fit. The model with two latent interaction effects has a LogL_Full_ = -9164.934, and the model without the interaction effect was LogL_Restricted_ = -9180.897. LR (*df* = 4) = 31.53, *p* < .001; the result suggest that including the interaction effects improved the overall model fit. In addition, the latent interaction effects of material hardship and resilience on internalizing and externalizing problems were not estimated simultaneously in the second model. This is because (a) the residual direct effects from material hardship to internalizing and externalizing problems were not significant in the mediating analysis, and (b) the model would become very complex and difficult to converge. Nonetheless, we conducted a supplementary analysis where only these interaction effects were included. The result indicated that adolescent resilience did not moderate the relationship between material hardship and internalizing problems (β = -0.06, *p* > .05) or the relationship between material hardship and externalizing problems (β = -0.11, *p* > .05).

**Fig 3:**
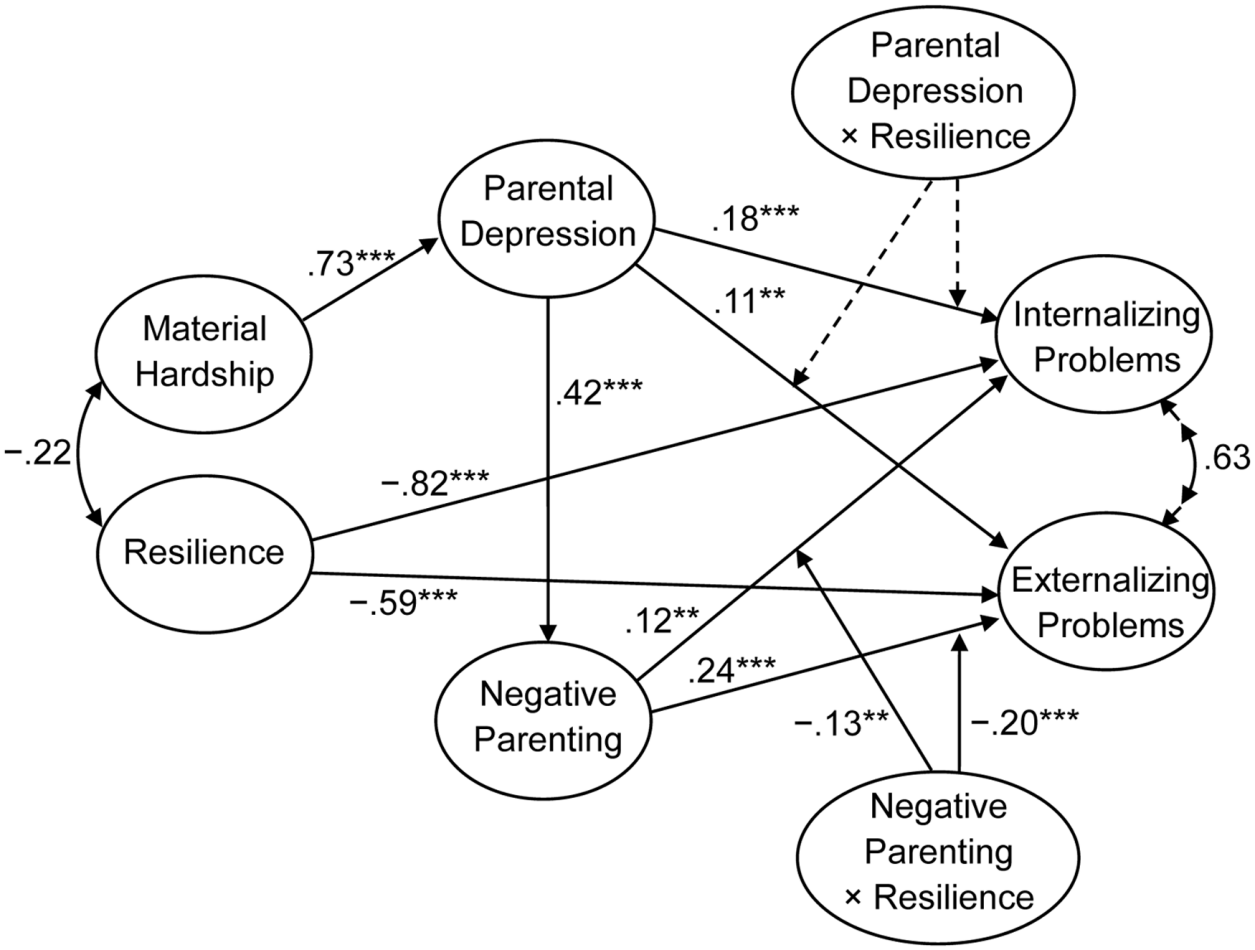
Unstandardized path estimates for structural model with latent interaction effects. Age, gender, primary caregiver, and family location were included as covariates in this model, but are not shown in this figure. Dashed lines represent nonsignificant latent interaction paths at *p* < .05. Curved arrows among latent variables represent correlated errors. **p < .01, ***p < .001.

As shown in Fig 3, negative parenting and resilience exhibited significant negative interaction effect in the prediction of both internalizing problems (β = -.13, *p* < .01) and externalizing problems (β = -.20, *p* < .01), thereby indicating that adolescents’ resilience served as a buffer that mitigates the adverse effects of negative parenting on problem behaviors. The latent interaction explained an additional 1% and 3% of the variance in internalizing problems and externalizing problems, respectively. However, contrary to our expectation, the interaction between parental depression and resilience did not significantly predict internalizing problems (β = .01, *p* > .05) or externalizing problems (β = .01, *p* > .05). These results partially supported our second hypothesis (Hypothesis B). In addition, considering readers may be interested in the separate moderating effects of the two high-order factors of the resilience, we also analyzed their effects on problem behaviors. The results were consistent with the moderating effects of total resilience.

**Fig 4:**
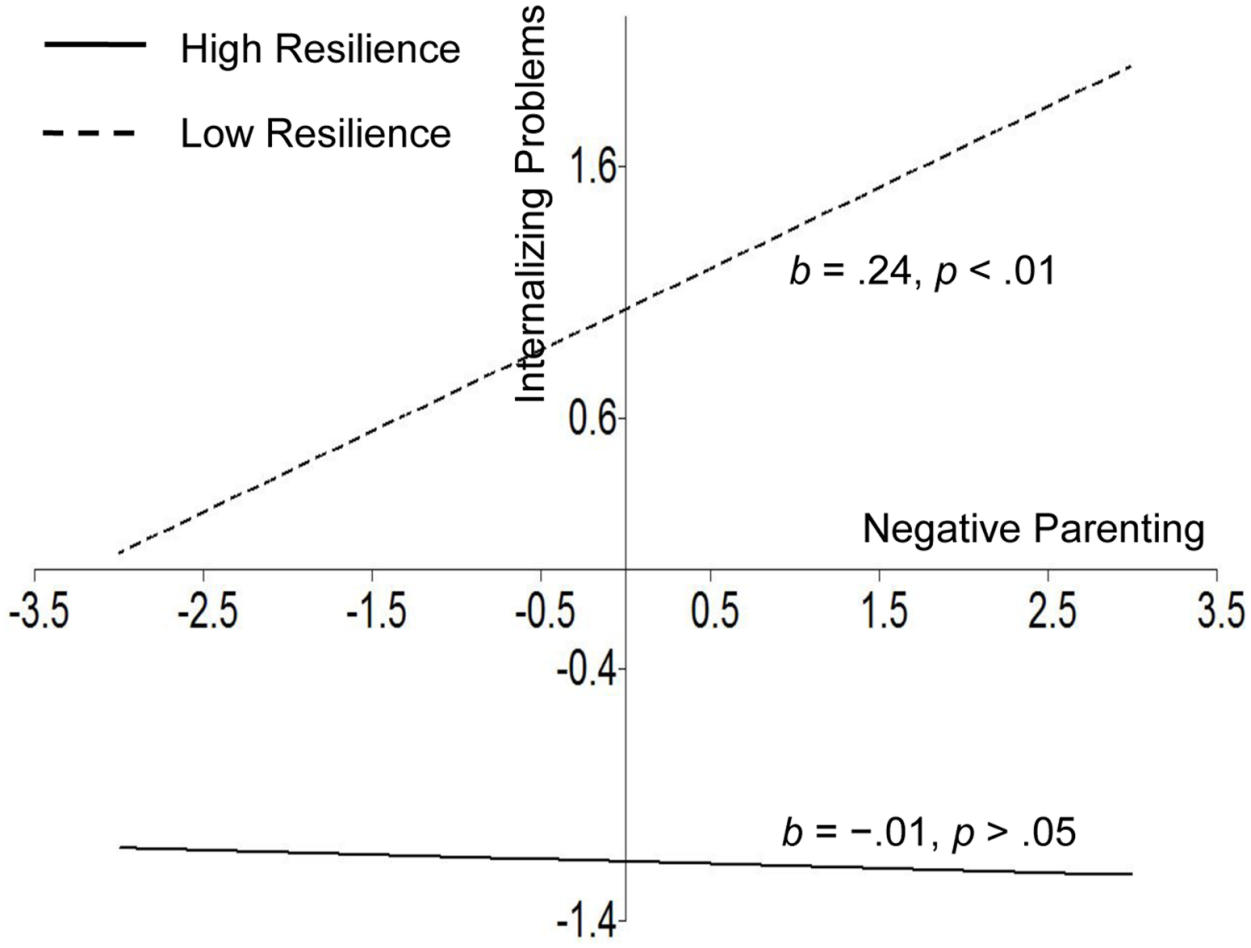
Resilience as a moderator between negative parenting and internalizing problems that illustrates the protective-stabilizing pattern.

**Fig 5:**
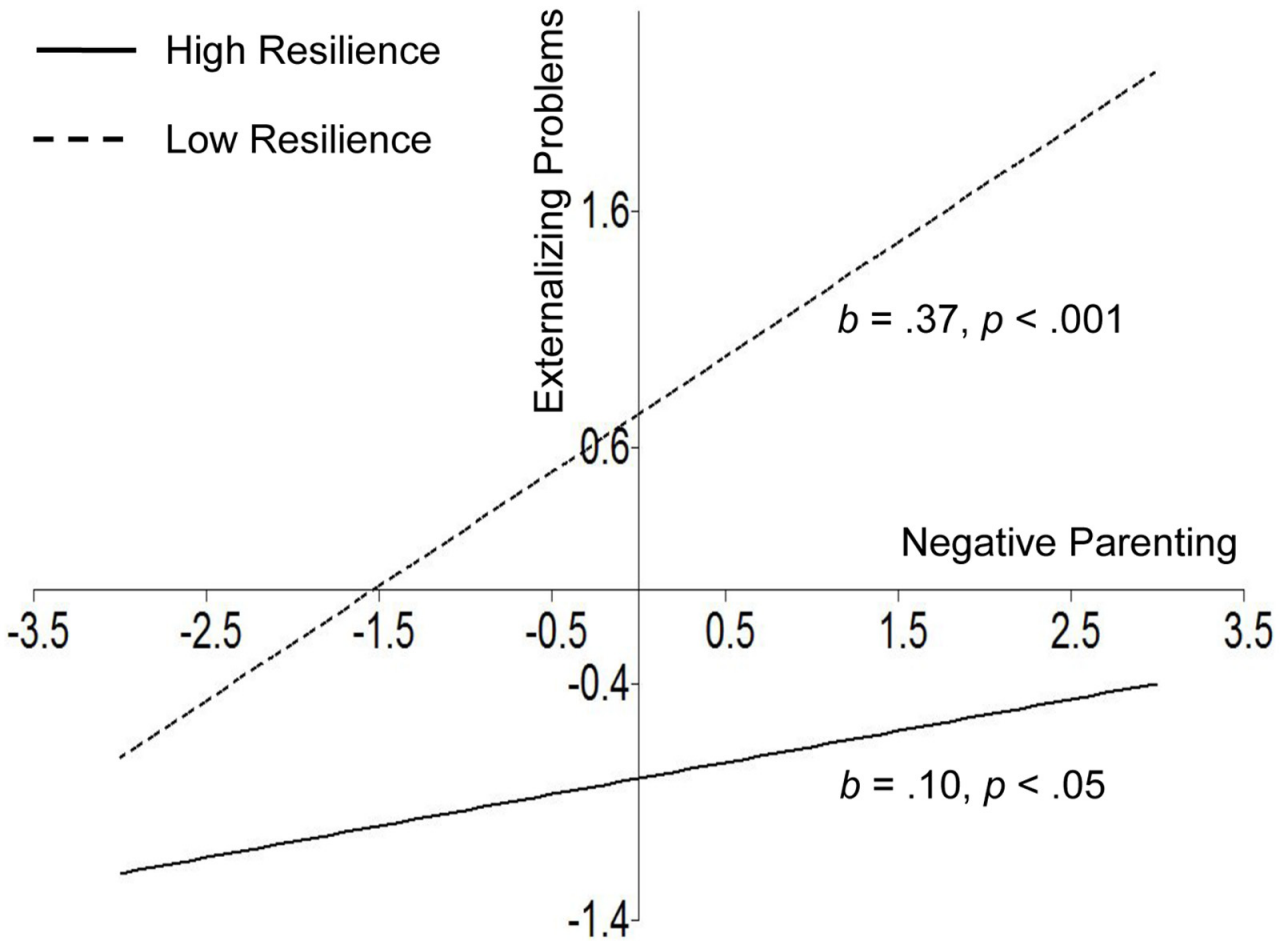
Resilience as a moderator between negative parenting and externalizing problems that illustrates the protective-reactive pattern.

For descriptive purposes, we used the procedures outlined by Muthén and Muthén (2012) in Mplus 7 to plot the predicted outcome variable by levels of the independent variable (range from -3 *SD* to +3 *SD*) at the high and low levels of the moderator (1 *SD* above the mean and 1 *SD* below the mean, respectively). Importantly, the latent variable indicators of internalizing problems and externalizing problems were centered in the moderation analysis; therefore, their values of them may be negative in Figs 4 and 5. As shown in Fig 4, for adolescents with low resilience, higher negative parenting was significantly associated with higher internalizing problems (simple slope = .24, *p* < .01). However, negative parenting was not significantly associated with internalizing problems for adolescents with high resilience (simple slope = -.01, *p* > .05). This result supported a protective-stabilizing pattern [13, 14]. Fig 5 shows externalizing problems as a function of negative parenting and adolescent resilience. The positive association between negative parenting and externalizing problems was smaller for adolescents with high resilience (simple slope = .10, *p* < .05) than for those with low resilience (simple slope = .37, *p* < .001). This finding supported a protective-reactive pattern [13, 14].

## Discussion

### The mediating effects of parental depression and negative parenting

The mediation analyses conducted herein supported the work of Conger and colleagues’ FSM [2, 11, 12] in a sample of Chinese adolescents and their parents. According to the FSM, economic strain is a grueling and demoralizing process that can lead to parents’ depressed mood. In turn, this distress negatively affects parenting; furthermore, less nurturing and involved parenting contributes to a host of psychological problems in children and adolescents [3, 68, 69]. In the present study, material hardship indirectly affected adolescents’ internalizing and externalizing problem behaviors through parental depression and negative parenting. That is, material hardship may have deteriorated parents’ mental health by increasing their depressive symptoms; in turn, high levels of depressive symptoms lead to more negative parenting (e.g., verbal hostility, physical coercion, and non-reasoning). This finding supports previous research that indicates that material hardship negatively influences children’s adjustment through its adverse influence on parental mental health and parenting behaviors [18, 24, 70].

There were no direct associations between material hardship and adolescents’ internalizing and externalizing problem behaviors. This may have been due to measurement issues; specifically, problem behaviors were self-reported by adolescents. Previous research has indicated that direct associations between economic disadvantage and children’s adjustment are commonly found when primary caregivers, teachers, clinicians, and peers are the informants about children’s mental health; however, when children report on their own mental health, indirect associations are often found, with paths between economic hardship and problem behaviors mediated through the actions of primary caregivers [68, 71].

We also found that parental depression fully mediated the relation between material hardship and negative parenting. This result was consistent with some previous research [24, 70]; however, other studies have not demonstrated the same pattern of results [17, 18]. This inconsistency in the literature may be due to the use of different measures in different studies. For example, Chien and Mistry’s study [70] included assessments of both positive parenting (e.g., parental warmth) and negative parenting (e.g., physical punishment). Although this requires further investigation, this finding highlights the critical role of parental depression in the relation between negative parenting and material hardship.

In addition, parental depression affected adolescents’ internalizing and externalizing problems both directly and indirectly through negative parenting; this result was consistent with previous studies [72, 73]. Furthermore, negative parenting was directly related to adolescents’ internalizing and externalizing problems [3, 74]. Taken together, the findings of the current study replicated the main tenets of the FSM in a sample of Chinese adolescents. Furthermore, the results of the current study add to a growing body of literature examining the influence of proximal family factors on the relation between material hardship and adolescents’ problem behaviors.

### The moderating effects of adolescent resilience

Previous studies on the FSM have focused on the mediating mechanisms that underlie the relation between material hardship and child problem behaviors; however, little attention has been paid to the heterogeneity of this relation [18, 68]. In fact, many adolescents experiencing material hardship did not have significant problem behaviors [7, 8]. Therefore, it is likely that individual assets (e.g., goal planning, affect control, and positive thinking) and external resources (e.g., family support and help-seeking) may mitigate the deleterious effects of material hardship on youth problems [13]. The present study examined adolescents’ resilience as a potential moderator of the relation between parental risk factors and adolescents’ problem behaviors. Our results suggest that adolescents’ resilience is a vital protective factor in the relationship between negative parenting and youth’s problems.

Consistent with previous research [31, 32], the present study indicated that resilience moderated the relationship between negative parenting and adolescents’ internalizing and externalizing problems. These findings indicated that individual development is complex and shaped by the interactions between the individual and environmental factors [75]. Specifically, a protective-stabilizing pattern of resilience emerged when examining the relation between negative parenting and internalizing problems; this finding suggests that negative parenting was not significantly associated with internalizing problems for adolescents with high levels of resilience. This result was consistent with previous studies [31]. In addition, a protective-reactive pattern of resilience was found when examining the relation between negative parenting and externalizing problems; this suggests that the effect of negative parenting on externalizing problems remained significant (although weaker) for adolescents with high levels of resilience. This result was also consistent with previous studies [32]. Taken together, these different interaction patterns suggest that the adverse impact of negative parenting on externalizing problems is less likely to be buffered by adolescent resilience than internalizing problems. This finding provides a deeper understanding of the mechanisms that underlie adolescent problem behaviors; in addition, this pattern of results also has important implications for the development of prevention and intervention programs for adolescents’ internalizing and externalizing problems.

Contrary to our expectation, adolescent resilience did not moderate the detrimental effects of parental depression on internalizing or externalizing problems. This suggests that the associations between parental depression and adolescent problem behaviors are more stable than the association between negative parenting and adolescent problem behaviors. This may be due to the fact that other factors influence the relation between parental depression and youth problem behaviors. For example, we did not examine the potential influence of heritability on the relation between parental depression and problem behaviors [72, 73]. Therefore, further research is needed to examine these relations.

### Limitations and future directions

There are several limitations and some caveats that need to be considered when interpreting the results of the present study. First, cross-sectional data were used; therefore, the direction of the results cannot be determined, and causality cannot be inferred. Indeed, it may be that some of the relations are bi-directional in nature [53]. For example, although the findings presented herein support the FSM, there are potential alternate explanations; namely, the findings may support the social selection view or interactionist approaches [see 3, for a review]. Therefore, future research should address this limitation to provide further evidence supporting the FSM. Second, although primary caregivers reported the family risks (material hardship, parental depression, and negative parenting) and adolescents reported their own problems (internalizing and externalizing problems), some of the relations between the variables may have been due to reporter bias (e.g., the correlations between material hardship and parental depression and the correlations between parental depression and negative parenting). Therefore, subsequent studies should use multiple raters and multiple methods of data collection. Third, the index of negative parenting was restricted to authoritarian parenting behaviors. Previous research suggests that poor and depressed parents are more likely to use authoritarian parenting practices [72, 76]; however, other parenting dimensions may also be relevant. Indeed, future studies should also include other parenting behaviors such as authoritative and permissive parenting. Fourth, the other parent who was not report on his/her depressive symptoms and negative parenting behaviors may also play an important role in the development of adolescents' problem behaviors; therefore, future study should examine simultaneously the roles of father’s and mother’s mental health and parenting behaviors on adolescents' problem behaviors. Finally, the findings presented herein cannot be generalized to clinical populations since the sample comprised a nonclinical population.

### Practical implications

Despite these limitations, this study has important practical implications. First, based on the findings of the FSM, the reduction of material hardship will likely considerably reduce parental distress and youth problem behaviors [6]. Second, the mediating mechanisms of parental risk suggest that intervention strategies that focus on reducing parental depression and improving positive parenting behaviors are likely to be effective in reducing adolescent problem behaviors. Indeed, many interventions targeted at children in low-income families have been designed to implicitly or explicitly focus on processes that underlie the link between poverty and poor developmental outcomes [77]. For example, Compas and colleagues implemented an intervention for parents with a history of major depressive disorder by increasing positive parenting practices; this intervention reduced children’s internalizing and externalizing symptoms [78]. Third, the present study suggests that adolescents’ resilience is a vital protective factor in mitigating the development of internalizing and externalizing problems. Therefore, interventions may need to focus on developing assets and resources for adolescents exposed to material hardship. This will likely be as important as reducing risk factors.

In conclusion, the current study merged the FSM and resilience theory to address how and when family material hardship impacts Chinese adolescents’ problem behaviors. The results validated the FSM in the context of Chinese culture. More importantly, the present study extended the FSM by examining whether adolescents’ resilience buffered the associations between parental risks and youth problem behaviors. These findings suggest that adolescents’ resilience is a vital protective factor that mitigates youth problem behaviors in the contexts of material hardship. The moderated mediation model tested herein contributes to a more comprehensive understanding of risk and resilience in youth development. Importantly, these findings have implications for the prevention of adolescent problem behaviors.

## Supporting Information

S1 File Data of the present study. (XLS)
